# Characteristics and factors influencing soil organic carbon composition by vegetation type in spoil heaps

**DOI:** 10.3389/fpls.2023.1240217

**Published:** 2023-10-12

**Authors:** Yiwen Yao, Quanhou Dai, Ruxue Gao, Xingsong Yi, Yong Wang, Zeyin Hu

**Affiliations:** ^1^Key Laboratory of Karst Georesources and Environment, Ministry of Education, Guizhou University, Guiyang, China; ^2^College of Forestry, Guizhou University, Guiyang, China; ^3^Institute of Soil Erosion and Ecological Restoration, Guizhou University, Guiyang, China

**Keywords:** vegetation type, soil organic carbon composition, spoil heaps, soil physical and chemical factors, fine root biomass

## Abstract

**Introduction:**

The variation of organic carbon content in spoil heaps is closely related to improving soil structure, maintaining soil fertility, and regulating soil carbon cycling balance. Analyzing the soil organic carbon content and related driving factors during the natural vegetation restoration process of spoil heaps is of great significance for promoting the accumulation of soil organic carbon in the spoil heaps.

**Methods:**

we selected spoil heaps with the same number of years of restoration to research the variations in soil organic carbon components under different vegetation types (grassland: GL, shrubland: SL, secondary forest: SF) and compared the results with those on bare land (BL).

**Results:**

Our results showed that vegetation type and soil depth significantly affect the content of soil organic carbon components. There was no difference in soil organic carbon components between SF and SL, but both were considerably superior to GL and BL (*p*<0.05), and the particulate organic carbon (POC) and light fraction organic carbon (LFOC) contents of SL were the highest. A significant positive linear correlation existed between SOC and active organic carbon components. Pearson’s correlation and redundancy analysis showed that the available potassium (AK) and total nitrogen (TN) contents and gravel content (GC) in the BL soil significantly impacted soil organic carbon. When vegetation is present, TN, total phosphorus (TP), and Fine root biomass (FRB) significantly affect soil organic carbon. Structural equation modelling (SEM) shows that AK and soil moisture content (SMC) directly affect the organic carbon composition content of BL, When there is vegetation cover, fine root biomass (FRB) had the largest total effect in the SEM. Soil bulk density (BD) has a negative impact on soil organic carbon, especially in the presence of vegetation.

**Conclusion:**

These findings suggest that vegetation restoration can significantly increase soil organic carbon content, FRB, AK, and TN play important roles in enhancing soil organic carbon. Supplementation with nitrogen and potassium should be considered in the bare land stage, and shrubs nitrogen-fixing functions and well-developed roots are more beneficial for the accumulation of soil organic carbon.

## 1 Introduction

The soil organic carbon (SOC) pool is one of the most important carbon pools in terrestrial ecosystems (Ahirwal et al., 2022), accounting for more than 50% of the total soil carbon (Dinakaran et al., 2022). Changes in soil organic carbon not only affect the nutrient supply of soils and vegetation and the carbon balance between soils and the atmosphere but also have a large impact on the Earth’s carbon cycle and global climate change (Lan, 2021; Liu et al., 2021). Furthermore, A higher SOC determines the structure and functionality of ecosystems, provides optimal soil conditions for plant growth, and promotes soil nutrient cycling (Schulte et al., 2014; Yang et al., 2020). It was found that vegetation type has a significant effect on soil organic carbon, and different vegetation types have different biomass accumulation and decomposition rates, which affect the input and output of soil organic carbon (Aliia et al., 2023; Angst et al., 2016).Therefore, knowledge of the composition and distribution of soil organic carbon under different vegetation types is important for assessing their local ecological impacts, such as mitigating climate change and enhancing ecosystem services and soil quality, especially in the context of the large number of spoil heaps (unnatural formation) generated by human activities in recent years.

With rapid economic development, population growth, and urban expansion, national and local government agencies have increased the construction of significant projects such as expressways, hydrological project, and airports to promote the rapid development of the economy (Xu et al., 2015; Xu and Liu, 2021). At the same time, these developments have brought on severe challenges for the surface environment (Hou et al., 2019). Infrastructure construction results in the production of many spoil heaps (Zhang et al., 2015). Data show that a total of 2.63×10^4^ soil and water conservation schemes were approved in China for production and construction from 2006 to 2015, and the cumulative disturbed surface area reached 15.64×10^4^ km^2^, among which the amount of discarded soil and slag was as high as 484.51×10^8^ m^3^ (Gao et al., 2018). The complex material composition, loose soil structure, poor cohesion, and poor water and fertilizer retention ability of construction spoil heaps readily lead to severe soil and water loss during rainfall events (Kaufman, 2000). Spoil heaps strongly disturb the original environment and restructure soils (Ahirwal and Maiti, 2018). Relevant studies have shown that when a soil layer is disturbed, the CO_2_ stored in soil pores is released into the atmosphere, which further exacerbates climate change (Zhang et al., 2019; Siegwart et al., 2022). Soil organic carbon is segregated into active organic carbon, slow organic carbon, and recalcitrant organic carbon fractions based on the turnover rate of soil carbon, among which active organic carbon includes particulate organic carbon (POC), easily oxidized organic carbon (EOC), microbial biomass carbon (MBC), light fraction organic carbon (LFOC) and soluble organic carbon (DOC) (Liu et al., 2021). Although it only accounts for a small amount of the total organic carbon, reactive organic carbon is an energy source for soil microbial activity and a driver of soil nutrient cycles, and it can directly participate in plant nutrient transformation and supply (Yu et al., 2017; Yang et al., 2020) and responds more rapidly to environmental changes than total organic carbon (Zhao et al., 2015). This has significant implications for accurately quantifying soil reactive organic carbon fractions to gain a deeper understanding of the soil carbon cycle (Wang and Wang, 2011). Therefore, carrying out research on soil organic carbon in construction spoil heaps can facilitate the management of the soil carbon pool and reduce carbon emissions from spoil heaps.

Vegetation not only serves as the main means of slope management and ecological restoration in construction spoil heaps (Cerqueira et al., 2012) but is also an important way to improve soil carbon sequestration (Maczkowiack et al., 2012; Xie et al., 2021). Vegetation affects and changes the content of soil carbon components through specific input and output processes based on organic matter input (Janna et al., 2017), plant root distribution (Angst et al., 2016), soil factors (Wang et al., 2023), and other environmental factors (Luo et al., 2022; Yu et al., 2021). The results showed that SOC accumulation was significantly different among vegetation types. The soil organic carbon in the grass-shrub mixed pattern increased by 85.1% compared to bare land (Wang et al., 2015). Liu et al. (2015) found in their research on the southwestern karst region that the organic carbon content in soil increased from 29.10 g·kg^−1^ in grassland to 73.92 g·kg^−1^ in native forests. Through a study of Ohio mine reclamation areas, Shrestha and Lal (2011) found that grassland is more conducive to the accumulation of carbon and nitrogen pools than forestland. In addition, through a study of a karst area in Southwest China, Liu et al. (2019) found that the content of soil organic carbon in a natural restoration model was significantly higher than that in an artificial restoration model and in cultivated land. However, since spoil heaps are organic structures with particular forms and functions that are different from those of natural ecosystems, their soil organic carbon distribution and cycling processes are also different from those of other ecosystems. Most studies on soil organic carbon and its active fraction have focused on piles such as those in coal mine drainage sites (Anderson et al., 2008; Ahirwal and Maiti, 2018) or have only considered a limited number of soil parameters involving topsoil. However, the soil organic carbon fraction is influenced by multiple factors, such as soil and vegetation (Luo et al., 2022), and cannot be analyzed based on individual factors. By these methods, it is impossible to distinguish between factors that play direct and indirect roles in determining the carbon fraction content. Therefore, it is necessary to investigate the changes that take place in the content of soil organic carbon fractions and related factors during the revegetation of spoil heaps.

In summary, current studies have confirmed the influence of vegetation on soil organic carbon fractions in spoil heaps. However, studies on the effect of vegetation restoration of spoil heaps on soil organic carbon fractions are still scarce. Therefore, our objectives were to (1) Understand the variation trend of soil organic carbon (SOC) content under different vegetation types in the spoil heaps. (2) Determine the key parameters that influence SOC during vegetation restoration process and explain how these parameters affect SOC. The results of this study can also help screen vegetation types suitable for the slope management of spoil heaps from the perspective of carbon sequestration capacity and improve our understanding of soil carbon sequestration during the vegetation restoration of spoil heaps.

## 2 Materials and methods

### 2.1 Study area description

**Figure 1:**
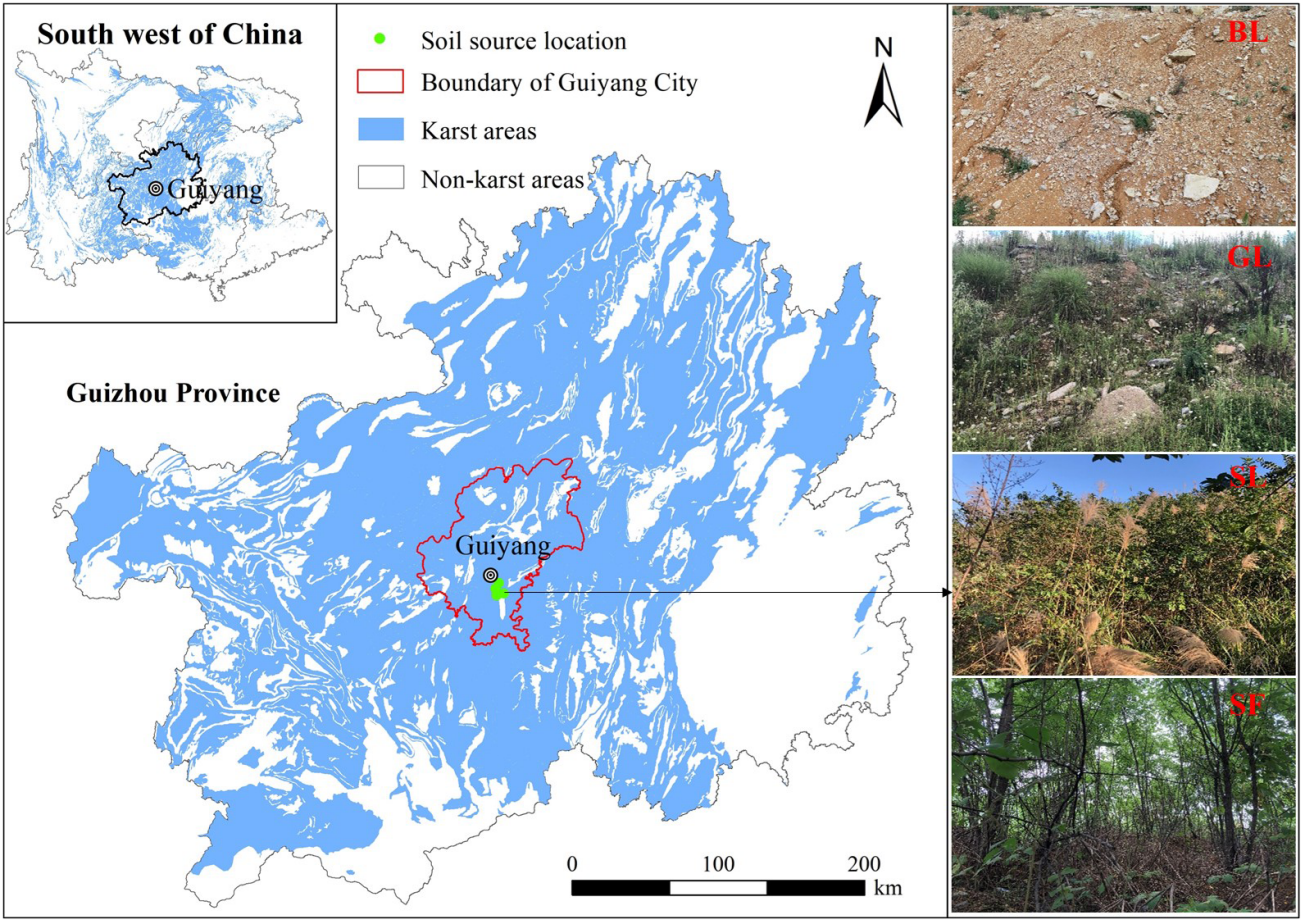
Geographical location map of the study area. BL represents bare land, GL represents grassland, SL represents shrubland and SF represents secondary forest.

The study area is located in Baiyun District, Guiyang City, Guizhou Province, Southwest China (106°35′E, 26°34′N) (**Figure 1**), which is a low-medium hilly area with shallow-cut denudation-type karst topography in the middle of the Guizhou Plateau. The annual average temperature is 15.3°C, the annual precipitation is 1085.5 mm, the annual sunshine duration is 1062.0 h, and the annual frost-free period is 270 days. The area has a subtropical humid monsoon climate, and the vegetation type is mainly subtropical evergreen broad-leaved forest. Due to the influence of human activities, the original vegetation of the spoil heaps area was destroyed. Soils were yellow soil developed from Quaternary red clay, which are classified as Luvisols according to World Reference Base for Soil Resources (Marquez et al., 1975). In addition, 103 construction spoil heaps generated by highway construction in Guizhou Province were investigated, and 35 of them were found to be slope-toppling types. The slope of these types of piles is mostly 30°~45°, and the proportion of soil and rock is between 25% and 40%. The elevation of the sample plot in this study is between 1252.06 and 1282.49 m, the slope is approximately 33°, and the gravel content is between 24.41% and 38.43%. We tried to ensure that the piles were essentially similar in slope, slope length, altitude, and other ecological factors to reduce influences from climate, topography, and other factors. The four most common vegetation restoration stages (BL, GL, SL, and SF) were selected for studying the construction spoil heaps. Specific information on the samples is listed in **Table 1**.

**Table 1:** Basic information of study plots.

Study sites	BL	GL	SL	SF
Coordinates	106°36′0.77″E 26°34′29.89″N	106°36′0.30″E 26°34′37.18″N	106°35′59.38″E 26°34′43.95″N	106°35′47.29″E 26°34′58.15″N
Average altitude (m)	1297.01	1297.01	1294.1	1282.49
Vegetation cover (%)	2	50-85	55-70	22-40
Average gravel content (%)	38.43	37.53	33.72	31.41
Average slope (°)	33	32	33	34.5
Slope aspect	NS	NS	NS	NS
Average slope length (m)	14.8	14.8	15	22
Soil type	yellow soil	yellow soil	yellow soil	yellow soil
Vegetation restoration mode	natural recovery	natural recovery	natural recovery	natural recovery
Vegetation features	Basically no vegetation growth	*Oxalis corniculate*, *Artemisia lavandulaefolia*, *Lolium perenne* and *Setaria viridis* are dominant species	*Coriaria nepalensis*, *Sophora flavescens*, *Rubus reflexus Ker* are dominant species, mixed with some herbs	*Robinia pseudoacacia* L. and *Populus* L. are the dominant species, while *Platanus acerifolia* and *Zelkova serrata* (Thunb.) Makino are partially distributed.
Disturbance history	The accumulation body was formed in 2010 and recovered naturally			

BL, bare land; GL, grassland; SL, shrubland; SF, secondary forest.

### 2.2 Experimental design and soil sampling

In September 2019, by using Google Earth historical image map, we preliminarily determined the geographical location and area of spoil heaps, and then determined the specific accumulation year of each spoil heap by field investigation, and selected four vegetation types in the same area, including bare land (BL), grassland (GL), shrubland (SL), and secondary forest (SF). Five plots (20 ×20 m) with representative structure and vegetation were selected for BL, GL, and SL. In comparison, three plots were established for SF due to the short recovery time of the pile and the limited number of plots that met the requirements. In order to reduce the impact of habitat heterogeneity on soil organic carbon components, the distance between sampling points should not be less than 100 meters. Five sampling points were selected from the diagonal of each plot. After litter was removed from the soil surface, a soil sampler (5 cm diameter) was used to sample the 0~10 cm (S), 10~20 cm (U1), 20~40 cm (U2), 40~60 cm (U3), and 60–80 cm (U4) layers. A total of 5 soil layers were collected, and a total of 450 soil samples were taken. In addition, during the soil sampling in each layer, soil was collected using a standard ring knife (200 cm^3^) for the measurement of soil bulk density and soil moisture content. This process was repeated three times per layer. During soil sampling, after debris such as stones and stumps were removed, each sample was divided into two parts. A portion of the fresh soil was stored in a refrigerator at 4°C after it was passed through a 2-mm sieve. The other portion of soil was naturally air-dried, placed in a marked bag after it was passed through a 0.15-mm sieve, and stored in an undisturbed and ventilated place (storage time was no more than one year) for the determination of soil properties.

Additionally, five points were randomly selected in every sample plot, and fine root samples were collected from the 0~10 cm, 10~20 cm, 20-40 cm, 40-60 cm, and 60-80 cm layers using a soil drill with an inner diameter of 7 cm. The collected samples were separately packaged and labeled and taken to the laboratory.

### 2.3 Soil analysis methods

The soil bulk density (BD) was sampled using a standard ring knife (200 cm^3^), and then the soil inside the ring knife was dried to constant weight at a high temperature of 105 °C (for at least 24 hours) and weighed, and the soil bulk density and soil moisture content (SMC) was calculated. The gravel content (GC)(>2 mm) was determined by sieve method (Zhan et al., 2020). The soil pH value was measured using the electrode method (soil water ratio 2.5:1) (Burgos et al., 2023). The total nitrogen (TN) content in soil was measured using the semi-micro Kelvin flow injection method. The total phosphorus (TP) content in soil was measured using NaOH melting molybdenum–antimony anti-chromogenic UV spectrophotometry. The total potassium (TK) content in the soil was determined using the NaOH melting atomic absorption method. The content of soil alkali hydrolyzed nitrogen (AN) was measured using the alkali hydrolyzed diffusion method. The content of soil available phosphorus (AP) was determined by NaHCO_3_ extraction–molybdenum–antimony anti-chromogenic ultraviolet spectrophotometry using 0.5 mol·L^-1^ NaHCO_3_ (Wu et al., 2018). The content of soil available potassium (AK) was determined by NH4Ac extraction atomic absorption spectrometry. Some indicator determination steps refer to the Chemistry Analysis of Agricultural Soil (Lu, 2000).

The root segments were immersed and rinsed in deionized water in the laboratory to remove soil and impurities attached to the root segments and the roots with diameter ≤ 2 mm (plant fine roots) were carefully picked up. At the same time, dead and living fine roots were distinguished according to the shape, epidermal color, elasticity and bending angle of fine roots. The main morphological parameters, such as root mean diameter, total length, total surface area, and total volume mean diameter, were obtained use the software WINRHIZO combined with EPSON Expression root analysis software (Paganová and Jureková, 2014). The scanned roots were dried at 70°C to a constant weight. The dry weight of fine roots was measured, and the biomass of fine roots per unit area was calculated (FRB, g·m^−2^). The following root parameters were determined: specific root length, (SRL, m·g^−1^) = root length/root dry mass; specific surface area (SSA, m^2^·g^−1^) = root surface area/root dry mass; root length density (RLD, m·m^−3^) = root length/soil core volume.

### 2.4 Soil carbon composition determination

Soil organic carbon (SOC) was determined using the potassium dichromate redox method (Bao, 2000).

For the determination of soil dissolved organic carbon (DOC), and 12.5 g soil was obtained by weighing appropriate amount of air-dried soil and passing through a 1 mm sieve. 50 mL K_2_SO_4_(0.5 mol/L) solution was added for leaching, and the filtrate was filtered after oscillating at 180 r/min on an oscillating machine for 30 min. The organic carbon in the filtrate was determined as DOC by TOC-VcpH organic carbon analyzer (Wang et al., 2019).

Soil particulate organic carbon (POC) was determined with reference to Wang et al. (2012) by modifying the method described by Cambardella and Elliott (1992). Firstly, an appropriate amount of air dried soil was weighed and sieved through a 2mm sieve to obtain 20.00 g of soil. 100 mL of sodium hexametaphosphate solution (5g/L) was added, and the soil suspension was obtained after shaking for 18 hours (90 rpm/min). The soil suspension was sieved through a 0.053mm sieve and repeatedly rinsed with distilled water. Then collect all the substances left in the sieve and bake at 60 °C for 48 hours to constant weight. Finally, the percentage of its content in the soil was calculated.

Soil microbial biomass carbon (MBC) was determined by chloroform fumigation-extraction method (Lin et al., 1999).Soil microbial biomass carbon was calculated by the difference between the fumigation sample and the control sample, MBC: Bc = Ec/Kec, where Ec is the difference between the fumigation and unfumigated soil, Kec is the conversion coefficient, and the value is 0.45.

Soil easily oxidized organic carbon (EOC) content was determined using the method of Blair et al. (1995). According to the volume ratio and concentration conversion relationship between the sample and the potassium permanganate solution, the content of easily *o*xidized organic carbon was calculated.

Soil light fraction organic carbon (LFOC) was determined by the modified density fractionation method (Jia et al., 2017).

### 2.5 Statistical analysis

The Tukey−Kramer method was used to analyze significant differences in soil carbon content and environmental factors under different vegetation restoration types. Multivariate analysis of variance (ANOVA) was used to analyze the effects of vegetation type and soil depth on soil organic carbon fractions. Pearson’s correlation analysis was used to analyze the correlation between soil organic carbon components, soil physical and chemical characterization, and fine root morphology. Partial correlation analysis was used to explore the relationship between soil physicochemical properties, vegetation roots, and soil organic carbon components. Redundancy analysis (RDA) was used to determine the relationship between soil physicochemical factors, fine root morphology, and soil organic carbon components. The forward screening method and Monte Carlo test were used to rank environmental factors to visually show the degree of contribution of explanatory variables to soil organic carbon components.

We used structural equation models (SEMs) to analyze the direct, indirect, and total effects of soil factors and vegetation roots on soil organic carbon composition. To increase degrees of freedom, we performed a Pearson correlation analysis between all predicted values in the model and removed indicators that weakly correlated with the SOC variable. The fitness of the final model was assessed using the model chi-squared test, root mean square error, and AIC. The SEM analyses were conducted using AMOS 21.0 (Amos Development Corporation, Chicago, IL, USA). RDA was performed using CANOCO5.0, and graphing was performed using Origin 2021.

## 3 Results

### 3.1 Changes in soil organic carbon components under different vegetation types

**Figure 2:**
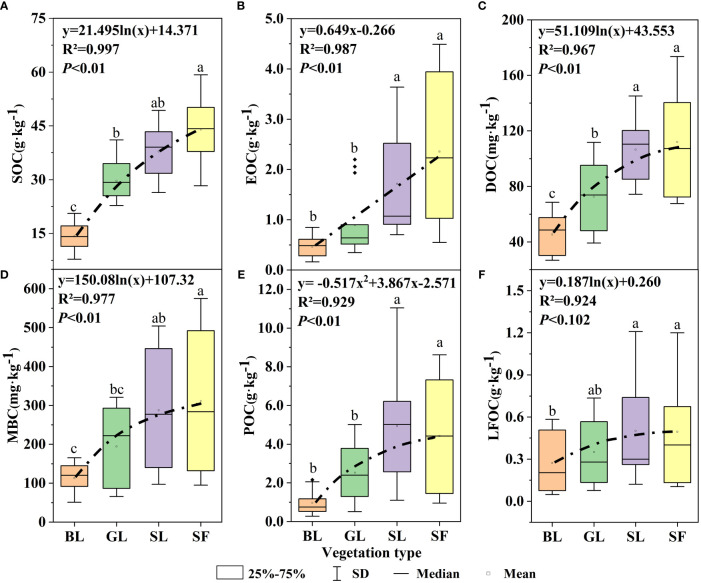
Changes in soil organic carbon components under different vegetation types. SOC, soil organic carbon; POC, particulate organic carbon; EOC, easily oxidized carbon; MBC, microbial biomass carbon; DOC, dissolved organic carbon; LFOC, light fraction organic carbon. The different letters on the boxplots indicate significant differences between different vegetation types (*p*<0.05).

During the vegetation restoration of spoil heaps, the SOC, DOC, EOC, and MBC of GL, SL and SF were significantly different from those of bare soil (*p*<0.05), but the difference in organic carbon components between SL and SF was not significant (**Figure 2**). The SOC content increased significantly from BL to SF (**Figure 2A**), with mean values of 14.17 g·kg^-1^, 29.70 g·kg^-1^, 37.89 g·kg^-1^, and 44.03 g·kg^-1^, respectively. There were significant differences in EOC and POC among SL, SF, BL, and GL (*p*<0.05) (**Figures 2B, E**). The EOC content of SF was five times that of BL, and the POC content of SL was 1.9 times that of GL. Compared with BL, the DOC of SF increased by 1.99 g·kg^-1^, and that of MBC increased by 200 mg·kg^-1^ (**Figures 2C, D**). The difference in the LFOC content among the three vegetation types was not noticeable (**Figure 2F**), but that of SL was the highest. The EOC content increased linearly with vegetation restoration (R^2 ^= 0.987 and *p*<0.01; **Figure 2B**), but the content of each organic carbon component increased with the progress of vegetation restoration in a logarithmic function (**Figure 2**).

**Figure 3:**
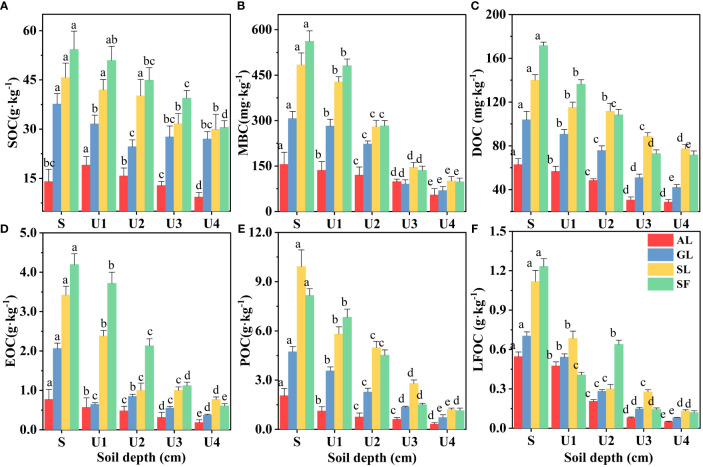
Changes in soil organic carbon content with soil depth. S: 0~10 cm; U1: 10~20 cm; U2: 20~40 cm; U3: 40~60 cm; U4: 60~80 cm. Different letters indicate that there are significant differences between vegetation types with different soil depths at the 0.05 level.

**Figure 4:**
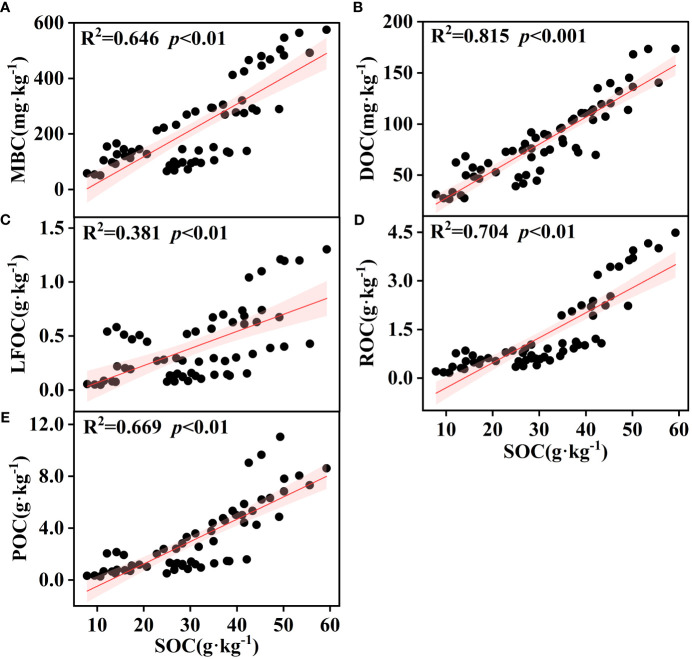
Simple linear regression analysis between soil SOC and various organic carbon components; the regression coefficients (R^2^) and *p* values are shown in the graphs. The red area represents the 95% confidence interval for the fit. SOC, soil organic carbon; POC, particulate organic carbon; EOC, easily oxidized carbon; MBC, microbial biomass carbon; DOC, dissolved organic carbon; LFOC, light fraction organic carbon.

The effect of soil depth on the content of various organic carbon components is shown in **Figure 3**. The soil organic carbon components showed apparent surface enrichment, and not all soil layers in SF had the highest content. When the soil layer depth exceeded that of the U2 (20~40 cm) layer, the MBC, DOC, POC, and LFOC of SL were higher. Linear fitting between soil organic carbon and its active components is shown in **Figure 4**. There was a significant positive correlation between the SOC of different vegetation types and DOC, MBC, LFOC, EOC, and POC (*p*<0.01). Among them, the SOC content was significantly positively correlated with that of DOC and POC (*p*<0.001). Two-way ANOVA showed significant associations between soil depth, vegetation type, and organic carbon components (EOC, DOC, MBC, POC, LFOC, and SOC) in all measured samples (**Table 2**). In addition, the content of each organic carbon component increased as a logarithmic function of the progress of vegetation restoration (**Figure 2**).

**Table 2:** Two-factor ANOVA testing the differences in soil organic carbon components.

Factor	SOC			POC			EOC		
	df	F	p	df	F	p	df	F	p
VT	3	294.235	0	3	443.89	0	3	658.754	0
SD	4	44.574	0	4	495.84	0	4	553.67	0
VT×SD	12	5.582	0	12	47.443	0	12	79.971	0
Factor	MBC			DOC			LFOC		
	df	F	p	df	F	p	df	F	p
VT	3	1236.75	0	3	756.15	0	3	189.207	0
SD	4	1982.06	0	4	450.64	0	4	1158.12	0
VT×SD	12	168.764	0	12	22.654	0	12	68.683	0

VT, vegetation type, SD, soil depth; SOC, soil organic carbon; POC, particulate organic carbon; EOC, easily oxidized carbon; MBC, microbial biomass carbon; DOC, dissolved organic carbon; LFOC, light fraction organic carbon.

### 3.2 Effects of vegetation types on soil physicochemical factors and root characteristics

#### 3.2.1 Changes in soil physicochemical factors under different vegetation types

**Figure 5:**
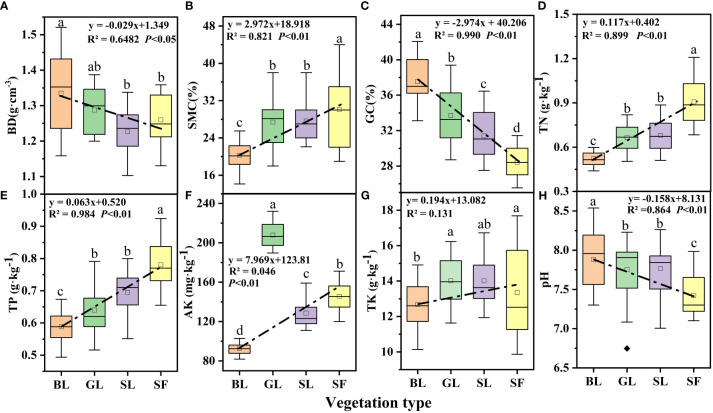
Soil physicochemical characteristics under different vegetation types. BL, bare land; GL, grassland; SL, shrubland; SF, secondary forest; BD, bulk density; SMC, soil moisture content; GC, gravel content (particle size>2 mm); TN, total nitrogen; TP, total phosphorus; AK, available potassium; TK, total potassium. The same notation is used in subsequent figures. The different letters on the boxplots indicate significant differences between different vegetation types (*p*<0.05).

Vegetation type significantly impacted soil factors, and the changes in soil physical and chemical factors under different vegetation types are shown in **Figure 5**. In general, BD decreased with vegetation restoration (**Figure 5A**) and that of BL was significantly higher than that of the other vegetation types (*p*<0.05). SMC showed an upward trend with vegetation restoration (R^2^ = 0.922, *p*<0.05; **Figure 5B**). The GC content was significantly and negatively correlated with vegetation restoration (R^2 ^= 0.990 and *p*<0.05; **Figure 5C**), and the GC content of SF decreased by 24.37% compared with that of bare land. Soil TN, TP, and AK all showed an increasing trend with vegetation restoration (**Figures 5D, E, F**), and vegetation restoration had a significant effect on all three factors (*p*<0.05). However, there was no difference between the TN and TP of GL and SL; AK content was the highest in GL, and pH was lowest in SF (**Figure 5H**).

**Figure 6:**
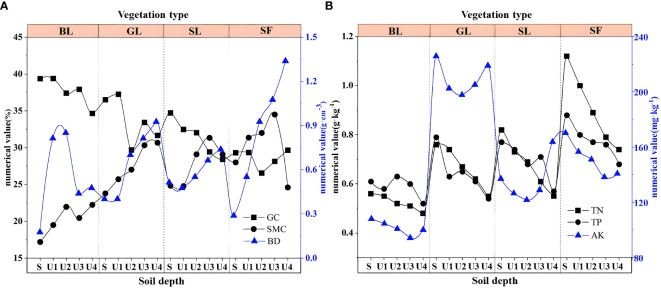
Soil physical and chemical properties at different depths. BL, bare land; GL, grassland; SL, shrubland; SF, secondary forest. S: 0~10 cm; U1: 10~20 cm; U2: 20~40 cm; U3: 40~60 cm; U4: 60~80 cm.

From the perspective of soil thickness, the physical and chemical indexes of the surface and deep soils showed relative changes. Among the soil physical indicators, the GC content decreased with increasing soil depth (**Figure 6A**), which may be related to the pores in the spoil heaps. The surface soil moves downward through the internal pores in the pile; as a result, the soil content is higher at the bottom than on the surface. Both BD and SMC increased with soil depth. The changes in soil TN, TP, and AK were roughly the same as those in the physical indicators, and all of these factors decreased significantly with increasing soil depth (**Figure 6B**).

#### 3.2.2 Root characteristics in different vegetation types

**Figure 7:**
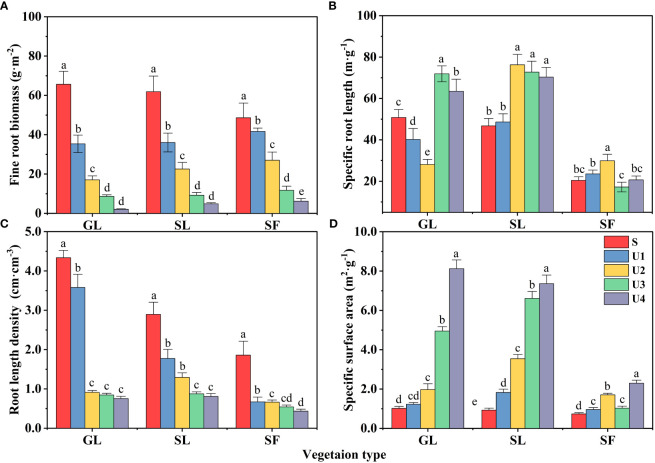
Fine root biomass and morphological characteristics. Different lowercase letters indicate significant differences between different soil depths for the same vegetation type (*p*<0.05).

Fine roots serve as the primary path for carbon to enter underground ecosystems. The vertical distribution pattern of fine roots largely determines the vertical distribution characteristics of soil organic carbon. The fine root biomass (FRB) of different vegetation types increased with the progress of vegetation restoration (**Figure 7A**) (*p*<0.05), and the lowest FRB was found in GL (128.76 g·m^-2^), followed by SL (134.55 g·m^-2^), and the highest was observed in SF (146.36 g·m^-2^). The FRB decreased significantly with increasing soil depth (*p*<0.05). The FRB of GL in the 0~20 cm soil layer accounted for 78.48% of the total soil biomass, that of SL for 72.76%, and that of SF for 68.64%. Among the different soil layers, the FRB of the surface (S) layer was dominant, showing apparent surface enrichment. In addition, plants may adapt to the nutrient and water supply patterns of spoil heaps by changing fine root distribution, morphology, and configuration. The results showed that there was no significant difference in SRL and SSA between SL and GL, but they were significantly higher than SF (*p*<0.05) (**Figures 7B, D**). SRL and SSA showed an upward trend with the increase of soil depth, and RLD gradually decreased with the increase of soil depth (**Figure 7C**). The decrease of GL content was the most obvious, with a decrease of 82.6%.

### 3.3 Correlation analysis of soil organic carbon components and related factors

**Figure 8:**
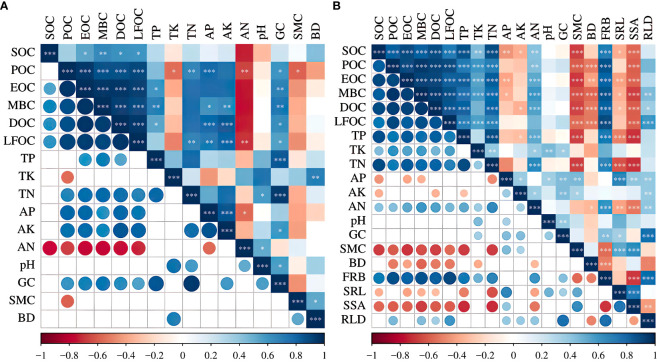
Pearson’s correlation analysis. * *p* < 0.05; ** *p* < 0.01; *** *p* < 0.001. SOC, soil organic carbon; POC, particulate organic carbon; EOC, easily oxidized carbon; MBC, microbial biomass carbon; DOC, dissolved organic carbon; LFOC, light fraction organic carbon; TN, total nitrogen; TP, total phosphorus; TK, total potassium; AN, available nitrogen; AP, available phosphorus; AK, available potassium. GC, gravel content; SMC, soil moisture content; BD, bulk density; FRB, fine root biomass; SRL, specific root length; SSA, specific surface area; RLD, root length density. Circle: the larger the correlation coefficient is, the larger the circle. The smaller the correlation coefficient is, the smaller the circle. Blue: the correlation coefficient is positive, and the more saturated the blue color is, the closer it is to 1.0. Red: the correlation coefficient is negative, and the more saturated the red color is, the closer it is to –1.0.

Pearson’s correlation analysis showed that before the establishment of vegetation (**Figure 8A**), the soil organic carbon content was significantly and positively correlated with soil TN, AP, AK, and GC and negatively correlated with soil TK, SMC, and AN, among which AN was significantly and negatively correlated (*p*<0.01). When vegetation cover was present (**Figure 8B**), soil organic carbon was significantly and positively correlated with the active organic carbon fractions, and the carbon fraction content was significantly and positively correlated with TP, TN, and FRB (*p*<0.01); negatively correlated with AP, GC, SMC and BD; and significantly and positively correlated with TK and AN (*p*<0.05).

**Figure 9:**
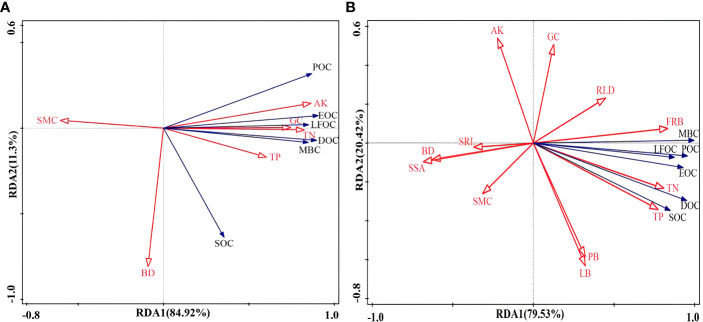
Redundancy analysis (RDA) ranking of soil physicochemical factors, root characteristics, and soil organic carbon components. **(A)** is for bare land, **(B)** is for soil with vegetation cover, and the meaning of the abbreviations is the same as that in **Figure 8**.

In addition, redundancy analysis was performed by screening out the indicators that were strongly correlated with the content of soil organic carbon components. RDA axis 1 (RDA1) and axis 2 (RDA2) explained 84.92% and 11.3% of the variation in soil organic carbon content without vegetation cover and 79.53% and 20.42% of the variation in soil organic carbon content with vegetation cover, respectively (**Figure 9**). The results showed that the contents of AK, TN, and GC in the bare soil significantly impacted soil organic carbon (**Figure 9A**). FRB, TN, and TP had significant effects on the organic carbon content when the soil−rock piles were covered by vegetation (**Figure 9B**). To more intuitively express the impact of explanatory variables on plant species diversity, the forward screening method and Monte Carlo test were used to rank environmental factors. Environmental factors with cumulative contribution rates greater than 80% were selected, and it was assumed that these factors played a significant role in influencing the content of soil organic carbon components under the conditions of each site (**Table 3**). The contribution rate of AK to the content of each organic carbon component in the bare land was the greatest, which was 64.1%, followed by the contribution rates of SMC, BD, and TP, which were 13.4%, 11.3%, and 9.7%, respectively. When vegetation cover was present, the contribution rate of FRB was the greatest (69.1%), and the cumulative contribution rate of TN, BD, and RLD reached 29.5%.

**Table 3:** Preliminary selection of environmental factors in RDA.

Type	Factor	Explains %	Contribution %	pseudo-F	P
Bare land	AK	49.7	64.1	12.8	0.002
	SMC	10.4	13.4	3.1	0.048
	BD	8.8	11.3	3.1	0.082
	TP	7.5	9.7	3.2	0.102
	GC	0.7	0.9	0.3	0.608
	TN	0.5	0.6	0.2	0.712
Vegetation	FRB	68.6	69.1	28.4	0.002
	TN	12	12.1	7.5	0.004
	BD	10.5	10.6	13	0.008
	RLD	3.5	3.5	6.5	0.014
	TP	3.3	3.3	14.1	0.004
	AK	0.5	0.5	2.7	0.126

**Figure 10:**
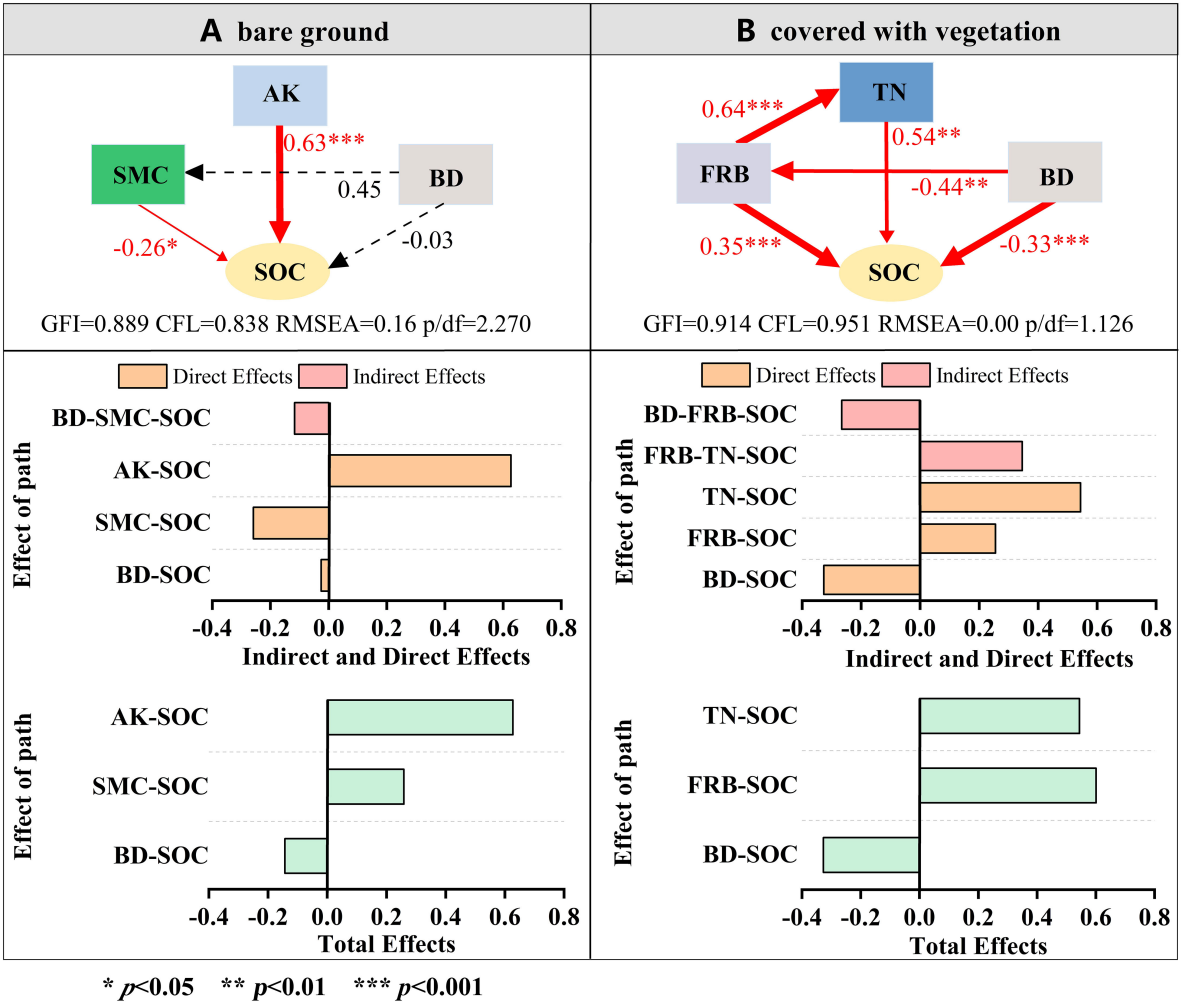
Structural equation model analysis of soil organic carbon variables. To make the model diagram more concise, the path coefficient from the control variable to the dependent variable is directly presented in the graph. Solid arrows indicate a significant effect (*p*<0.05), and a dashed arrow indicates a nonsignificant effect (*p*>0.05). The width of an arrow is proportional to the intensity.

To further explore how soil indicators and roots affect soil organic carbon fractions, we selected the top three contributing factors and used a structural equation model to explore the mechanism by which the factors affected soil organic carbon fractions. SEM analysis showed that in bare soil (**Figure 10A**), AK, the single and direct controlling factor that emerged from the final model, had a strong positive impact on soil active carbon content (standardized direct impact was 0.63), while BD and SMC both had adverse effects, and the standardized coefficients of direct impact were 0.03 and 0.26, respectively. After the establishment of vegetation (**Figure 10B**), TN and FRB directly affected the content of organic carbon (the direct effects of standardization were 0.54 and 0.35, respectively), and FRB also indirectly affected the content of organic carbon by affecting TN content. BD had a negative effect on the content of organic carbon components and indirectly affected the content of organic carbon components by affecting FRB. The total effect of SEM showed that in the absence of vegetation, AK and SMC had a significant positive effect on soil organic carbon components, and the effect of AK was higher than that of SMC. FRB had the most considerable total effect when vegetation was present, followed by TN. BD was negatively related to soil organic carbon components with and without vegetation on the spoil heaps, and the effect was more significant when there was vegetation.

## 4 Discussion

### 4.1 Effects of vegetation types on soil organic carbon and its components

Soil organic carbon content results from a net balance between the organic matter input rate and organic carbon mineralization rate (Sheng et al., 2015), and organic matter input is mainly determined by vegetation cover and plant roots (Yzab et al., 2021). A shift in vegetation type can significantly affect the balance between soil carbon input and output, changing the soil organic carbon content (Luo et al., 2022). With the gradual restoration of vegetation on mixed soil and rock construction piles, the apoplastic and plant root inputs under each vegetation type were significantly higher than those of bare ground, and SL and SF were significantly better than GL at increasing the content of the soil organic carbon fraction, which is consistent with the results of Zhang et al. (2019) but somewhat different from the results of Liu et al. (2021) for the Haidaigou coal mine drainage site. The latter study showed that the soil organic carbon content was in the order grassland > shrubland > secondary forest, which may have been caused by the fact that the restoration time of the pile selected in the present study was 9 years, while the study by Liu et al. was conducted on a coal mine drainage site with a restoration time of 15 years. The accumulation of soil organic carbon in the underground soil of arbor forests can be divided into two stages: the first stage is when the organic carbon content increases with the growth of plants. In this stage, many extraneous substances are returned to the surface soil every year, which provides energy sources for microorganisms, can accelerate the decomposition rate of humus, and increases the input of soil organic carbon (Zechmeister-Boltenstern et al., 2015; Ghimire et al., 2019). Therefore, the average soil organic carbon content of secondary forest was higher; however, the content of POC was the highest in SL. The dominant species in SL was mulberry, and the leaves of mulberry wither quickly, the thickness of the surface apoplast is higher and the surface layer is wetter compared to that of SF and BL, and a large amount of plant residues are formed, which contribute semidecomposable material; as a result, the agglomerated particulate matter increases, and the organic carbon embedded in the particulate matter increases accordingly (Xiao et al., 2021; Yuan et al., 2022). In bare land, due to the absence of vegetation cover, the soil remains exposed for long periods, soil particles are separated due to sunlight exposure, the soil aggregate structure is destroyed, soil organic carbon remains unprotected (Yang et al., 2020), the soil is loosened due to anthropogenic disturbance, and a breakdown in aggregates may lead to the loss of unstable POC and EOC, which are mineralized to CO_2_.

In this study, during vegetation restoration, soil organic carbon recovery in the S layer was much greater than that in the other soil layers because organic carbon inputs from arbor forests and shrubs mainly contributed to the aboveground litter (Ghimire et al., 2019; Blackburn et al., 2022). The surface layer of the soil of the spoil heaps had large amounts of plant apoplasts, and the humus formed from apoplast decomposition mostly remained in the surface soil. The surface soil structure facilitated water permeability and aeration, which accelerated the decomposition rate of apoplasts and reduced the loss of soil nutrients while increasing the accumulation of organic carbon (King and Hofmockel, 2017). However, the organic carbon content of the bare land surface layer was not the highest. The survey showed that SL and SF average vegetation covered 30% to 60%, and bare land was not covered by vegetation (**Table 1**). Rainfall leads to the mechanical removal of soil organic carbon due to runoff on slope surfaces, and the erosive force of rainfall impact causes large soil agglomerates to break apart, causing the organic carbon that was originally protected by the agglomerates to be decomposed and used by microorganisms, leading to a loss of soil organic carbon in the surface layer of bare land. This is consistent with Mma et al. (2020), who showed that changes in vegetation type resulted in an average loss of 7% in soil carbon, with the loss concentrated in the topsoil, and the deep soil organic carbon content was less affected, which is consistent with the results of this study. Subsoil organic carbon is mainly derived from root secretions, soluble organic carbon leaching, soil fragmentation, and particulate organic carbon transport, and the carbon input generated is limited (Lv and Liang, 2012). Most of the root biomass is concentrated in the 0-40 cm soil layer, and plant roots and their secretions decrease with increasing soil layer depth (Luo et al., 2022). The soil environment becomes closed, the input of organic matter and microbial activity diminish, and the effect of vegetation on SOC gradually decreases. This also explains the nonsignificant difference in organic carbon content under the vegetation types in the U3 and U4 layers.

### 4.2 Response of soil vegetation factors to vegetation types

Vegetation affects soil carbon distribution by changing soil physical and chemical properties and microbial activities. Soil physical and chemical indexes are significantly different under different vegetation types (Bakker et al., 2019; Zhou et al., 2022). The soil BD and GC gradually decreased with positive vegetation succession. As succession progresses, the biological activity of vegetation increases, leading to accelerated accumulation and decomposition of organic matter (Yan et al., 2019). In addition, penetration, extinction and biological activities of root systems help to stabilize soil particles, reduce soil looseness, and improve soil pore characteristics, which make soil more loose and are conducive to the growth of roots and microorganisms (Bengough et al., 2011), thereby reducing soil bulk density and gravel content. This is consistent with the findings of Bengough et al. (2011) and Lu et al. (2014). Nevertheless, soil SMC was lower in SF than in GL and SL (**Figure 6**) because these vegetation types had shallower root systems and smaller canopies relative to SF, allowing for less transpiration and water consumption. Moreover, the average litter thickness of GL and SL (2.5 cm) was higher than that of SF (2 cm), and the accumulation of litter also reduced water loss to a certain extent (Lee et al., 2014). Soil TN and TP showed an increasing trend with the restoration of vegetation (**Figure 6**), indicating a significant effect of vegetation on soil quality improvement.

Vegetation returns to the soil during growth, development and death, forming soil organic matter through humification and providing a source of nutrients after mineralization and decomposition (Tanner et al., 2016; Yang et al., 2020). Soil BD, GC, and SMC all showed a downward trend with increasing soil depth (**Figure 7**), consistent with the results of Zhang et al. (2019). The surface soil was more strongly affected, the evaporation of soil water on the surface was more significant, and the water content was lower. FRB was the highest in SF and low in GL, which is consistent with the findings of most studies (Hishi et al., 2015; Wang et al., 2020), but surface FRB dominated total fine root biomass, and FRB was significantly higher in GL than in SL and SF (**Figure 8**). Because the roots of herbs such as *Lolium perenne* and *Setaria viridis* in this study are primarily shallow root–fibrous root systems, the proportion of fine surface roots was relatively large. In the deep layer (U3 and U4), the FRB of SF was higher than that of GL and SL because to absorb more water and nutrient sources for their growth and development, the roots of tree species such as *Robinia pseudoacacia* had to extend downward. Hence, the root biomass in the deep soil was higher.

### 4.3 Analysis of factors influencing soil carbon components

The organic carbon content of soil is influenced by various external factors, such as microbial decomposition, soil acidity and alkalinity, and soil erosion, and is closely related to the internal carbon content of soil (Wei et al., 2019; Ferro and Nicosia, 2020). In addition, research results have shown that soil organic carbon is the main determinant of active organic carbon, and there is a highly significant positive correlation between different active organic carbon components, indicating that they are closely related and jointly affect the turnover of soil organic carbon as similar active carbon pools (Wang et al., 2020). However, in this study, there was no significant correlation between soil SOC and POC under bare ground conditions (**Figure 9A**), which differs from the results of previous studies, potentially due to the external damage sustained by the bare ground soils of the spoil heaps and due to the destruction of agglomerates during soil separation, dissipation, and fragmentation, which allows the organic carbon within agglomerates to be exposed, accelerating POC decomposition (Wu et al., 2023). In addition to directly affecting the content and distribution of SOC, vegetation also affects SOC indirectly by influencing environmental factors related to SOC formation and transformation. We investigated the effects of soil factors and vegetation roots on the organic carbon fraction under two site conditions—with and without vegetation cover species—on the soil surface of soil–rock construction spoil heaps. It was found that soil organic carbon and active carbon fractions were significantly and positively correlated with TN in both bare ground and soils with vegetation cover because most of the N in the soil is stored in organic matter (Xie et al., 2020), and increased nitrogen in soil may inhibit soil respiration and thereby reduce the amount of CO_2_ released from soil (Andrew et al., 2018). However, abundant nitrogen supply can enhance microbial activity, promote the decomposition by surface microorganisms, and increase the content of organic carbon and its active carbon fraction (Liu et al., 2021; Xu et al., 2020), which explains the significant positive correlation between the soil organic carbon fraction and total nitrogen content in construction spoil heaps.

Soil texture is considered to be an important factor affecting soil organic carbon accumulation (Sebastian et al., 2023), and studies have shown that clay has a greater SOC storage potential than sandy soil (Wiesmeier et al., 2019; Kgel-Knabner and Amelung, 2021), which ascribe to the physical chemistry adsorption of SOC on soil clay mineral surfaces, SOC is chemically stabilized (Cai et al., 2016; Ji et al., 2020). In this study, due to the loose soil, large sand particles, small relative surface area and strong water permeability, organic carbon in the sand is easily decomposed or lost by microorganisms. Due to the high gravel content in bare land, Water-soluble potassium is released from minerals within the mound in the presence of bacteria and various acids that can further increase organic carbon content by influencing microbial activity (Bakken et al., 1997). When vegetation was present, FRB was the most influential factor on the content of organic carbon and its components, with a contribution of 69.1%. Guo and Gifford (2002) and Davidson et al. (2011) suggested that root distribution and quality are key determinants of soil organic carbon response to vegetation type because plants transport organic matter to the subsurface through root secretion and abscission, can use their own biomass inputs to modify organic carbon content (Zhou et al., 2022), and can also increase inputs to soil organic carbon sources through their interactions with certain symbiotic bacteria (Wei et al., 2019). Roots can either directly affect SOC or affect SOC and its components by affecting TN content, showing the highest total effect in the structural equation model (**Figure 10B**), which also confirms the root contribution to soil SOC. Studies have shown that the same factors contribute differently to organic carbon under different environmental conditions, and the dominant factors affecting organic carbon accumulation change according to the prevalent conditions. In addition, plant-derived carbon input is an important factor affecting soil organic carbon and its components. Although deadfall is an important supplemental channel for supplying carbon, it also leads to microorganism proliferation; as a result, a large amount of soil enzymes are produced, which accelerate the decomposition rate of soil organic carbon, resulting in a decrease in organic carbon content (Wang et al., 2020; Erdel et al., 2023). Therefore, the effect of plant-derived carbon such as litter on soil organic carbon and its components needs to be studied in depth.

Therefore, according to the results of this study, nitrogen and potassium supplementation should be considered during the bare land stage to enhance organic carbon accumulation in spoil heaps. From the perspective of carbon sequestration efficiency and prevegetation investment, it is reasonable to prioritize the planting of shrubs with nitrogen fixation functions and well-developed root systems on spoil heaps.

## 5 Conclusions

Vegetation not only increased soil organic carbon content but also improved soil physical and chemical properties during the restoration process in spoil heaps. There was no significant difference in the content of soil organic carbon between secondary forest and shrubland, and some of them were higher in shrubland (POC and LFOC), indicating that in the early stage of formation of the spoil heaps, the selection of secondary forest may not be able to better improve the content of soil organic carbon components. The content of the soil organic carbon fraction in the 0-20 cm layer under each vegetation type was significantly higher than that in the other soil layers. There was a significant positive correlation between soil organic carbon and active carbon components under various vegetation types, indicating that active organic carbon, which has a short turnover time, can be used as an indicator to evaluate the impact of vegetation type changes on the soil organic carbon pool. FRB, AK and TN played important roles in increasing soil organic carbon, and nitrogen and potassium supplementation should be considered in the bare land stage. Prioritizing the planting of shrubs with nitrogen fixation functions and well-developed roots is beneficial for soil organic carbon accumulation.

## Data availability statement

The original contributions presented in the study are included in the article/supplementary material. Further inquiries can be directed to the corresponding author.

## Author contributions

YY and QD: conception and design of the research. YY and XY: acquisition of data.
